# Structuring Nonlinear Wavefront Emitted from Monolayer Transition-Metal Dichalcogenides

**DOI:** 10.34133/2020/9085782

**Published:** 2020-04-05

**Authors:** Xuanmiao Hong, Guangwei Hu, Wenchao Zhao, Kai Wang, Shang Sun, Rui Zhu, Jing Wu, Weiwei Liu, Kian Ping Loh, Andrew Thye Shen Wee, Bing Wang, Andrea Alù, Cheng-Wei Qiu, Peixiang Lu

**Affiliations:** ^1^Wuhan National Laboratory for Optoelectronics and School of Physics, Huazhong University of Science and Technology, Wuhan 430074, China; ^2^Department of Electrical and Computer Engineering, National University of Singapore, 4 Engineering Drive 3, Singapore 117583; ^3^Advanced Science Research Center, City University of New York, New York 10031, USA; ^4^Department of Physics, National University of Singapore, 2 Science Drive 3, Singapore 117542; ^5^Institute of Materials Research and Engineering, Agency for Science, Technology and Research, 2 Fusionopolis Way, Innovis, #08-03, Singapore 138634; ^6^Department of Chemistry, National University of Singapore, 3 Science Drive 3, Singapore 17543; ^7^Centre for Advanced 2D Materials, National University of Singapore, Block S14, 6 Science Drive 2, Singapore 117546; ^8^Hubei Key Laboratory of Optical Information and Pattern Recognition, Wuhan Institute of Technology, Wuhan 430205, China

## Abstract

The growing demand for tailored nonlinearity calls for a structure with unusual phase discontinuity that allows the realization of nonlinear optical chirality, holographic imaging, and nonlinear wavefront control. Transition-metal dichalcogenide (TMDC) monolayers offer giant optical nonlinearity within a few-angstrom thickness, but limitations in optical absorption and domain size impose restriction on wavefront control of nonlinear emissions using classical light sources. In contrast, noble metal-based plasmonic nanosieves support giant field enhancements and precise nonlinear phase control, with hundred-nanometer pixel-level resolution; however, they suffer from intrinsically weak nonlinear susceptibility. Here, we report a multifunctional nonlinear interface by integrating TMDC monolayers with plasmonic nanosieves, yielding drastically different nonlinear functionalities that cannot be accessed by either constituent. Such a hybrid nonlinear interface allows second-harmonic (SH) orbital angular momentum (OAM) generation, beam steering, versatile polarization control, and holograms, with an effective SH nonlinearity *χ*^(2)^ of ~25 nm/V. This designer platform synergizes the TMDC monolayer and plasmonic nanosieves to empower tunable geometric phases and large field enhancement, paving the way toward multifunctional and ultracompact nonlinear optical devices.

## 1. Introduction

In the past decade, nonlinear signals (second- and third-harmonic generation, SHG and THG) from nanomaterials and artificially structured materials, namely, metamaterials and metasurfaces, have attracted extensive attention for their wavelength tunability, coherence, and ultrafast responses [1–6]. These materials show promising applications in nanoprobing [1], imaging [2, 3], and crystalline detection [4–6]. Compared with the control of linear optical signals with a metasurface [7–14], the wavefront engineering and control of generated nonlinear signals are the most challenging task to realizing integrated, ultrathin, and efficient nonlinear optical devices, ranging from nonlinear beam steering [15, 16], nonlinear metalenses [17, 18], nonlinear holography [19, 20], optical image encoding [21], and the generation of a nonlinear optical vortex beam [22–24]. There have been several pioneering works on nonlinear phase control based on plasmonic metasurfaces, most of which focus on SHG manipulation [17, 20–27]. However, their weak second-order effective susceptibility, due to the inherently weak nonlinear response [22, 28] of plasmonic materials that rely on surface symmetry breaking, poses an intrinsic challenge for broad applications. Although SHG can be boosted by orders of magnitude to the level of ~pm/V under resonance conditions, the samples may be easily damaged by thermal effects due to the strong absorption. Alternatively, nonlinear hybrid metasurfaces, such as Au/multiple quantum well (MQW), have been proposed as an alternative to traditional plasmonic metasurfaces [29–31]. Previous works report 2~3 orders of magnitude of enhancement following this route [32–35]. Nevertheless, demanding fabrication requirements make it difficult to design ultrathin and compact multifunctional nonlinear metasurfaces that are needed for the flexible wavefront control of nonlinear signals.

On the other hand, emerging 2D materials, such as transition-metal dichalcogenide (TMDC) monolayers, are well known for their strong light-matter interactions and valley-polarized optoelectronic properties [36–44]. Of particular interest is the large nonlinearity of monolayer TMDCs (with chemical composition *AB*_2_, *A* = Mo, W, Ta, and Nb and *B* = S, Se, and Te). Their broken centrosymmetry enables a second-order susceptibility *χ*^(2)^ around 1~100 nm/V [45–48], which is much larger than the one of many plasmonic metasurfaces (see Section 3 in SI for details), and is even comparable to record values reported in Au/MQW hybrid metasurfaces (~54 nm/V) [29]. However, the polycrystalline nature of most CVD-grown TMDC monolayers is only several tens of *μ*m in size; it is quite challenging to spatially control the amplitude, polarization, phase, and even angular momentum of the emitted SH from monolayer TMDC. A paradigm-shift mechanism is needed to boost, manipulate, and control the SH photons of atomically thin materials.

Nature actually provides a hint towards such a mechanism. Epoxy is a type of strong glue made from two resins, with neither individually able to make the glue sticky. Putting those two resins together, a strong and powerful adhesive is obtained, because a chemical bond is produced. Inspired by the working principle of epoxy, we experimentally report a recipe for full control of SH photons via an integrated nonlinear optical interface composed of plasmonic nanosieves strongly coupled to a TMDC monolayer. As a proof-of-concept validation, TOC illustrates the schematic of bonding a WS_2_ monolayer with a plasmonic Au nanosieve; the bonded assembly behaves as a hybrid nonlinear optical interface for multifunctional SHG manipulation. SHG is in principle forbidden in Au nanosieves since they are made of centrosymmetric rectangular nanoholes, but the integrated hybrid optical interface offers highly efficient SHG emission, whose properties may be precisely controlled at a hundred-nanometer pixel-level resolution. SHG, originating from the WS_2_ monolayer, is enhanced by the strong local fields within each Au nanoholes, and simultaneously, the geometric phase determined by individual Au nanoholes can be locally engineered and imparted to the WS_2_ monolayer on top of the Au nanosieves. Our experimental results indicate that an effective SHG susceptibility of ~25 nm/V is achieved, 3 orders of magnitude larger than the one of conventional plasmonic metasurfaces [49]. Based on these principles, we demonstrate in the following several multifunctional nonlinear optical devices for free-space transmissive nonlinear optical interfaces for beam steering, versatile polarization control, orbital angular momentum (OAM) generation, and holograms. This strategy readily leads to the realization of a plethora of designer functions, enabling a new platform, i.e., TMDC-metasurface nonlinear optical interfaces, to realize robust nonlinear applications (e.g., high-order harmonic generation, HHG) with high efficiency, ultracompactness, and flexible fabrication.

**Figure 1:**
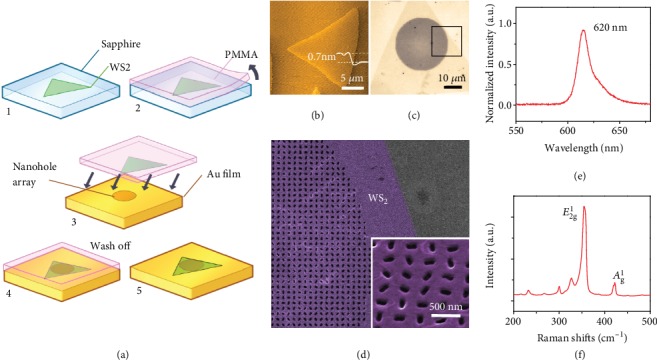
(a) Schematic for transferring WS_2_ monolayers onto a gold nanosieve by the PMMA-assisted transfer method; (b) AFM image of a WS_2_ monolayer with a height profile of ~0.7 nm. (c) Optical image of the Au-WS_2_ interface. The Au metasurface is fully covered by a triangular WS_2_ monolayer. (e, f) TPL and Raman spectra from a WS_2_ monolayer, respectively.

Figure 1(a) illustrates the fabrication processing of a monolayer Au-WS_2_ metasurface, which can be easily prepared by a material transferring method (see Methods for details). An atomic force microscope (AFM) image of the WS_2_ flake shown in Figure 1(b), whose height profile is measured to be ~0.7 nm, indicates a WS_2_ monolayer. An optical image of the WS_2_ monolayer on the Au nanosieve is shown in Figure 1(c), indicating that the Au nanosieve is fully covered by a triangular WS_2_ monolayer with an area of 100 *μ*m^2^. Figure 1(d) presents a scanning electron microscope (SEM) image of the same sample, indicated by the square area in Figure 1(c), where the high-quality WS_2_ monolayer can be clearly observed. Figure 1(e) presents the two-photon induced photoluminescence (TPL) spectrum from a WS_2_ monolayer under the excitation at 810 nm, showing the peak wavelength at ~620 nm. Figure 1(f) shows the Raman spectrum of the WS_2_ monolayer excited at 532 nm. Two typical signatures located at 352.3 and 417.3 cm^−1^ can be observed, which are attributed to the *E*_2g_^1^ and *A*_g_^1^ phonon modes from the WS_2_ monolayer, respectively. In general, the characterization results prove the high quality of the WS_2_ monolayer, and their intrinsic properties are not changed in the hybrid Au-WS_2_ interface.

**Figure 2:**
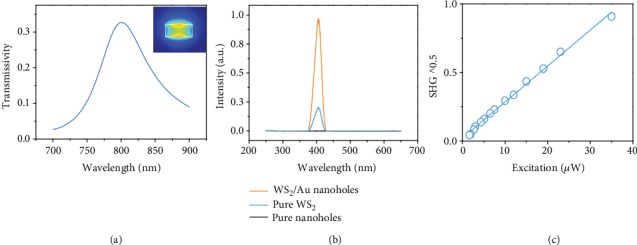
(a) Calculated transmission spectrum of a single Au rectangular nanohole under a CP excitation by using the FDTD method. The inset shows the calculated electric-field distribution at 810 nm. (b) Measured spectra of the emitted SH signals of the Au-WS_2_ interface (red curve), a bare WS_2_ monolayer (blue curve), and pure Au nanoholes (black curve). (c) Intensity of the emitted signal of the Au-WS_2_ interface increases linearly as the square of incident power.

Figure 2(a) shows the calculated transmission spectrum of a single Au rectangular nanohole under a circular polarized (CP) excitation by using the FDTD method. The inset shows the calculated electric-field distribution at 810 nm, which is in good agreement with the wavelength of the fundamental laser at 810 nm. Figure 2(b) presents the measured spectra of the emitted SH signals of the Au-WS_2_ interface (red curve), a bare WS_2_ monolayer (blue curve) and pure Au nanoholes (black curve). It can be clearly seen that both curves have sharp peaks at 405 nm, which is the SHG wavelength. Note that no SHG can be observed in the bare Au metasurface under the same excitation (black curve). As shown in Figure 2(c), the intensity of the emitted signal of the Au-WS_2_ interface is increased linearly as the square of incident power. Thus, it can be concluded that the emitted signal is ascribed to the SHG of the WS_2_ monolayer. More importantly, the measured SHG intensity from the Au-WS_2_ interface is 5 times stronger than the one from the pure WS_2_ monolayer under the same excitation condition, which is ascribed to the strong field confinement in Au nanoholes. The area ratio of the nanohole and the whole unit cell is around 10%. Without considering the local-field enhancement effect, the SHG intensity of the Au-WS_2_ interface should be 10%^2^ = 0.01 in comparison with that of a pure WS_2_ monolayer. Therefore, the SHG intensity in the area of nanoholes of the Au-WS_2_ interface is estimated to be enhanced by two orders of magnitude (5/0.01 = 500 times). Previous work indicates that the WS_2_ monolayer has a large second-order susceptibility of ~5 nm/V [35], and the effective second-order susceptibility of the Au-WS_2_ interface is estimated to be ~25 nm/V which is three orders of magnitude larger than the one of typical plasmonic metasurfaces (~pm/V).

**Figure 3:**
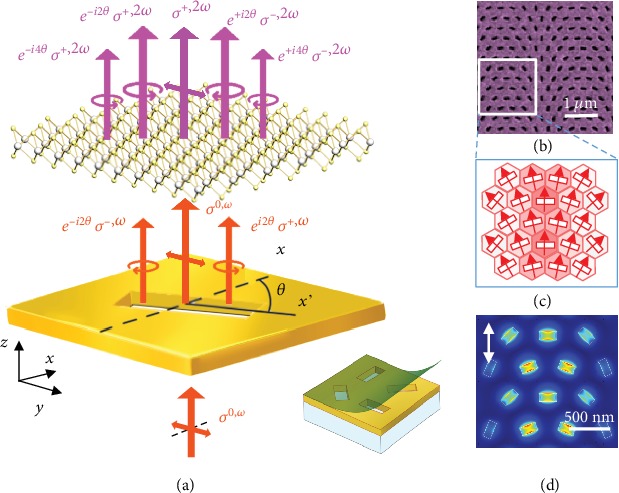
(a) Illustration of the principle of SHG phase control in the Au-WS_2_ interface. (b) SEM image for linear plasmonic nanosieves with the gradient phase. (c) Polarization distributions (indicated by the arrows) of the fundamental beam passed through the Au nanohole array in the area indicated by the square in (b). The deeper color indicates stronger local fields in the Au nanoholes. (d) The calculated local-field distributions in Au nanoholes of the same area by the FDTD method.

The underlying physics of the SHG phase control in the integrated Au-WS_2_ interface is illustrated in Figure 3(a). It refers to a pixel for generating an SH signal with phase information. For a single Au nanohole, the rotation angle, *θ*, is defined as the angle between the long axis of the rectangular nanohole and the *x*-axis. The nanohole can be considered as an ideal polaroid with an extinction ratio  *t*_*x*_ ≫ *t*_*y*_, and letting *t*_*x*_ = 1, the Jones' matrix can be calculated by

(1) $$\begin{array}{c} J\left(\theta \right)=R\left(-\theta \right)\left[\begin{array}{cc} 1 & 0\\ 0 & 0 \end{array}\right]R\left(\theta \right), \end{array}$$

where *θ* is the rotation angle of a nanohole and

(2) $$\begin{array}{c} R\left(\theta \right)=\left(\begin{array}{cc} \cos\theta & \sin\theta \\ -\sin\theta & \cos\theta \end{array}\right), \end{array}$$

is the rotation matrix. When a linearly polarized (LP) fundamental beam, *σ*^(0, *ω*)^, a *y*-axis polarized

(3) $$\begin{array}{c} \left[\begin{array}{c} 1\\ 0 \end{array}\right], \end{array}$$

is normally incident on the rectangular Au nanohole, the transmitted beam will be

(4) $$\begin{array}{c} \left[\begin{array}{c} E_{x}\left(\omega \right)\\ E_{y}\left(\omega \right) \end{array}\right]=\left[\begin{array}{c} \cos\left(\theta \right)\\ \sin\left(\theta \right) \end{array}\right]\cos\left(\theta \right), \end{array}$$

then it can be decomposed into

(5) $$\begin{array}{c} \left[\begin{array}{c} E_{x}\left(\omega \right)\\ E_{y}\left(\omega \right) \end{array}\right]=\frac{1}{4}\left\{2\left[\begin{array}{c} 1\\ 0 \end{array}\right]+e^{i2\theta }\left[\begin{array}{c} 1\\ -i \end{array}\right]+e^{-i2\theta }\left[\begin{array}{c} 1\\ i \end{array}\right]\right\}. \end{array}$$

It indicates that the transmitted beam can be decomposed into three components: left-handed circularly polarized (LCP, *σ*_−_) and right-handed circularly polarized (RCP, *σ*_+_) beams, with geometric phase discontinuity ±2*θ*, and a LP beam without geometric phase, which are written as *e*^(∓*i*2*θ*)^, *σ*^(±,*ω*)^, and *σ*^(0, *ω*)^, accordingly. For the WS_2_ monolayer with the $D_{3\text{h}}\;\left(\bar{6}m2\right)$ point group symmetry, and assuming that the symmetry axis of the WS_2_ monolayer is parallel to the *x*-axis, the second-harmonic susceptibility is *χ*_*xxx*_^(2)^ = −*χ*_*xyy*_^(2)^ = −*χ*_*yxy*_^(2)^ = −*χ*_*yyx*_^(2)^ = *χ*^(2)^, and we have

(6) $$\begin{array}{c} E_{x}\left(2\omega \right)\propto \chi^{\left(2\right)}\left\{{\left[E_{x}\left(\omega \right)\right]}^{2}-{\left[E_{y}\left(\omega \right)\right]}^{2}\right\},\\ E_{y}\left(2\omega \right)\propto 2\chi_{xyx}^{\left(2\right)}E_{x}\left(\omega \right)E_{y}\left(\omega \right). \end{array}$$

And then it can be decomposed into

(7) $$\begin{array}{c} \left[\begin{array}{c} E_{x}\left(2\omega \right)\\ E_{y}\left(2\omega \right) \end{array}\right]=\frac{1}{8}\left\{\left(e^{-i4\theta }+2e^{-i2\theta }\right)\left[\begin{array}{c} 1\\ -i \end{array}\right]+2\left[\begin{array}{c} 1\\ 0 \end{array}\right]+\left(e^{i4\theta }+2e^{i2\theta }\right)\left[\begin{array}{c} 1\\ i \end{array}\right]\right\}. \end{array}$$

As these fundamental beams further pump the WS_2_ monolayer, the emitted SH from the WS_2_ monolayer/Au nanohole has five components with different phase jump and polarization. The 0^th^-order SHG beam is a LP beam without deflection (along the *z*-axis). The ±1^st^-order and ±2^nd^-order deflected beams are CP beams (LCP and RCP) with a phase delay of ∓2*θ* and ∓4*θ*, respectively. Due to the conservation of angular momentum, these deflected SHG beams have a polarization state orthogonal to the excitation fundamental beams. Therefore, the ±1^st^-order and ±2^nd^-order deflected beams can be written as *e*^(±2*iθ*)^ *σ*^(±,2*ω*)^ and *e*^(±4*iθ*)^ *σ*^(±,2*ω*)^, respectively. The SHG phase can therefore be precisely controlled by orientating the Au nanoholes, which are also entangled with a spin angular momentum (SAM). Note that the intensity of the ±1^st^-order deflected beams is much stronger than the one of the ±2^nd^-order beams, so we mainly focus on the manipulation of ±1^st^-order SHG beams in the following. We stress that the results as well as its underlying mechanisms are fundamentally different from conventional nonlinear metasurfaces made of symmetric nanostructures (see Sections 1 and 2 in SI for further discussions), suggesting that this approach of hybridizing linear plasmonic metasurfaces with monolayer semiconductors with giant nonlinearity offers a new frontier in plasmonic metasurfaces. Therefore, we can realize multifunctional SH wavefront controls by designing an Au metasurface with a specific phase information.

Next, we elaborate on how this strategy can be implemented to realize multifunctional nonlinear optical devices. To demonstrate SHG manipulation, we designed a linear plasmonic nanosieve with a gradient phase as shown in the SEM image in Figure 3(b). The array is formed by a patterned rectangular Au nanohole with varying orientations, realizing a photonic spin-Hall metasurface with a geometric phase gradient (*∇φ*) along the *x*-axis [13, 50]. A hexagonal arrangement with a period of 400 nm is employed to increase the density of unit cells. Specifically, Figure 3(c) shows the polarization distribution of the fundamental beam as it passes through the Au nanohole array (indicated by the rectangle area in Figure 3(b)), which is predominantly polarized along the short axis of each nanohole (indicated by the arrows). The deeper color indicates a stronger local field in the Au nanohole, which is further confirmed by FDTD simulation results in the same area shown in Figure 3(d).

**Figure 4:**
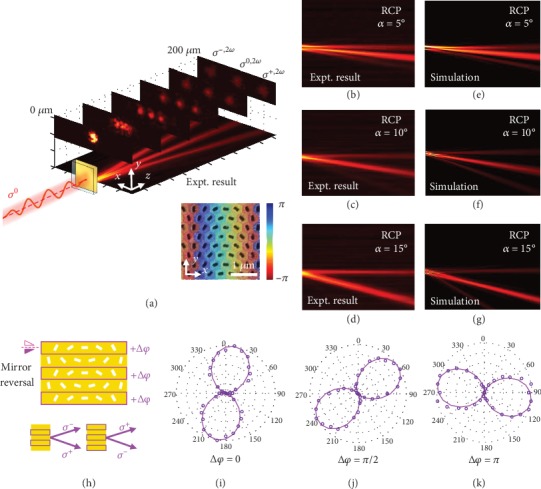
(a) Experimentally measured propagation of an emitted SH signal along the *z*-axis under a LP excitation (polarized along the *y*-axis). The inset shows the phase distribution of the SH signals, analog to a typical photonic spin-Hall metasurface along the *x*-axis. The inset images captured along different positions of the *z*-axis present the emitted SH spots. (b–d) Measured RCP components from three samples with different deflected angles of 5°, 10° and 15°, respectively. (e–g) The numerical simulated results corresponded with the experimental results in Figures 4(b)–4(d), respectively. (h) Schematic of the versatile control of the polarization of the deflected LP beams. (i–k) Polarimetric plots of SHG intensity from samples with phase differences of *d*sin(*α*) = 0*λ*, 0.25*λ*, and 0.5*λ*, respectively.

In the inset of Figure 4(a), we show the phase distribution of the SH signals of the sample shown in Figure 3(b). Figure 4(a) presents the measured propagation of emitted SHG along the *z*-axis. It can be seen clearly that the emitted SH signals are split into three beams. The inset images captured at different positions along the *z*-axis present a clear illustration of the SHG spots, indicating a low divergence angle of the SHG beams. Specifically, the 0^th^-order SHG beam propagates along the *z*-axis without any deflection due to the fundamental laser not carrying a geometric phase, while the rest travel along two sides of the *z*-axis with the same deflection angle (±1^st^ order). The deflection angle *α* is measured to be 10°, in good agreement with the theoretical design (10°). Furthermore, the polarization states of the SH beams are analyzed. The Stokes parameters *S*_3_(*S*_3_ = (*I*_RCP_ − *I*_LCP_)/(*I*_RCP_ + *I*_LCP_)) of -1-, 0-, and 1-order SH beams are determined to be -0.90, -0.32, and 0.95, respectively. This indicates that the ±1^st^-order deflected beams have opposite CP with almost pure polarization, while the 0^th^-order SH beam is approximately LP. In Figures 4(b)–4(d), we show the extracted RCP component from the experimental results of different samples with designed deflection angles of 5°, 10°, and 15°, respectively. Correspondingly, the deflected RCP beams are observed at *α* = 5°, 10°, and 15°, while the RCP component of the 0^th^-order beam propagates without deflection and the LCP component is blocked by a quarter-wave plate coupled with a Glan-laser polarizer. Interestingly, a weak 2^nd^-order SH beam can also be observed with a deflection angle of ~10° (Figure 4b), which is approximately twice the design angle of 5°. However, this cannot be observed in Figures 4(c) and 4(d), which may be ascribed to a low diffraction efficiency at a large deflection angle and limited detection efficiency in our experiment. Figures 4(e)–4(g) present that the numerical simulated results corresponded with the experimental results in Figures 4(b)–4(d), respectively, which is in good agreement with the experimental results (see Figure S2 in SI for further discussions).

Following the beam steering results, two deflected SHG beams propagate along different sides of the *z*-axis with opposite spin angular momentums (SAMs). By reversing the order of the supercells arranged on the Au nanosieve, the SAM of the deflected SHG beams can be reversed, while keeping the same deflection angle. As a result, this integrated interface can generate a LP beam by a superposition of two SHG beams with opposite SAMs, whose polarization can be arbitrarily controlled by adjusting the phase difference of the two SHG beams.  Figure 4(h) illustrates the schematic of versatile polarization control of the deflected SHG beam under excitation with a LP fundamental laser (*y*-axis polarized). Specifically, by reversing the direction of the phase gradient of the supercells arranged on the odd lines of the Au metasurface, half are converted to deflect an opposite SAM while keeping the same deflection angle (*α* = 10°), thus generating a LP beam. Note that both deflected LP beams have the same polarization. The phase difference of *σ*^+,2*ω*^ and *σ*^−,2*ω*^ can be controlled by adjusting the optical path difference, *d*sin(*α*), where *d* is the offset of the odd and even lines of the metasurfaces with an inversed order of the supercells. Figures 4(i)–4(k) present the polarimetric plots of the measured SHG intensity as a function of the polarization angle, *β*, at phase differences, *d*sin(*α*) = 0*λ*, 0.25*λ*, and 0.5*λ*, respectively. *β* = 0° implies that the SHG beam is linearly polarized along the *y*-axis. All measured SHG beams are approximately purely linearly polarized. The polarization directions for each beam are indicated by the long axis of the patterns at 10°, 55°, and 100°, respectively. This is in good agreement with the design rule *β* = ((*d*sin(*α*))/*λ*)*π*, where *λ* is the SHG wavelength. We find the same deviation angle of 10° for each SHG beam, which is attributed to the orientation of the WS_2_ monolayer [29]. This is quite different from the polarization control in the linear case [41]. Such versatile nonlinear polarization generation can find applications in commercial polarization modulators [51].

**Figure 5:**
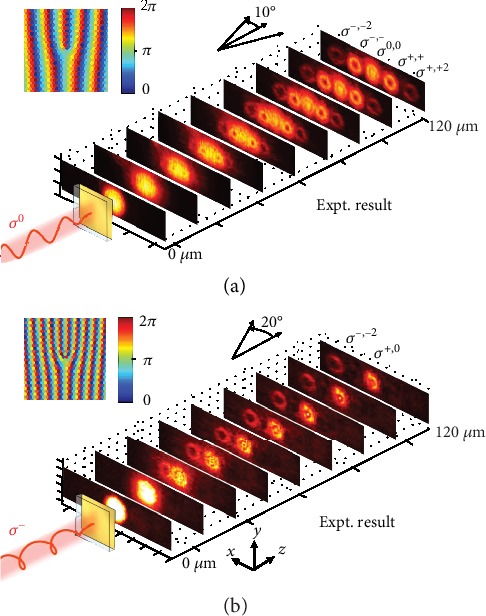
(a) Measured spatial intensity (log) profile of emitted SHG beams. The inset indicates the SHG phase profile of the sample for OAM generation under the LP fundamental beam (polarized along the *y*-axis). (b) Measured spatial intensity profile of emitted SHG beams. The inset indicates the SHG phase profile of the same sample under the CP fundamental beam.

The inset of Figure 5(a) presents the phase distribution of the 1^st^-order RCP component of the SH emission from each pixel under a LP pumping (*y*-axis polarized), which can be described as

(8) $$\begin{array}{c} \frac{x\sin\left(\alpha \right)}{\lambda }+\frac{n\gamma }{2\pi }, \end{array}$$

where *x* is the coordinates of the nanoholes, *λ* is the wavelength of SHG, *α* is the deflection angle, *n* is the topological charge, and *γ* is the azimuth angle in a circular coordinate system. Here, the deflection angle is *α* = 10° and the geometric topological charge is *n* = 1. In Figure 5(a), we show the measured spatial intensity profiles of the emitted SH signals under a LP fundamental beam (*y*-axis polarized), and five SHG beams are clearly seen as predicted by our theory. Specifically, the 0^th^-order beam with a circular spot is the LP beam without deflection. The ±1^st^-order SH beams are propagated along both sides of the *z*-axis with the same deflection angle, indicating a doughnut-shaped spot due to the properties of OAM beam propagation. Both beams have opposite CP with the same value of deflection angle, *α* = 10°, which is consistent with our design. Importantly, the topological charge is determined to be ±1 by the interference pattern between the 0^th^-order and ±1^st^-order beams (the details for the interference patterns are described in Figures S3 and S4 in SI). Note that both ±2^nd^-order SHG beams present a thinner doughnut-shaped pattern with a much weaker intensity than the ±1^th^-order SHG beams, whose topological charge is ±2 in theory. The deflection angle is measured to be around 20°, which is twice that of the ±1^st^-order beams under the small deflection angle approximation.

Interestingly, the phase plate of SH emission of the same sample appears to be quite different under the excitation of a CP fundamental beam, as shown in the inset of Figure 5(b), since the phase delay is doubled under CP excitation as compared to LP excitation. Figure 5(b) shows only two SHG beams: the SHG beam along the *z*-axis is LP without deflection, similar to the 0^th^ order in Figure 5(a). The -1^st^-order deflected SHG with a deflection angle of 20°, whose intensity distribution is doughnut-shaped with a topological charge of -2, is consistent with theoretical expectations. The spot shape, polarization, deflection angle, and topological charge of the -1^st^-order deflected SHG beam are the same as the -2^nd^-order SHG beam under LP excitation shown in Figure 5(a). Such difference can be attributed to the dependence of SHG on the amplitude of the fundamental beam. Our demonstration of dynamic nonlinear OAM generation under excitation with different polarizations can serve as the nonlinear version of the recently demonstrated metasurface-enabled quantum entanglement of SAM and OAM [52]. The change of the spatial deflection angle of the -1^st^ order in different pumping polarizations, i.e., resulting in different spots in the image plane, can also be further explored to realize a nonlinear quantum metasurface for photon state reconstruction [53]. Thus, our demonstration of spin-entangled and spatially tunable nonlinear OAM here becomes very powerful for free-space quantum imaging, generation of entangled photon states, and other applications. Overall, these results provide clear evidence of the hundred-nanometer-precision phase control of SHG from the Au-WS_2_ interface.

**Figure 6:**
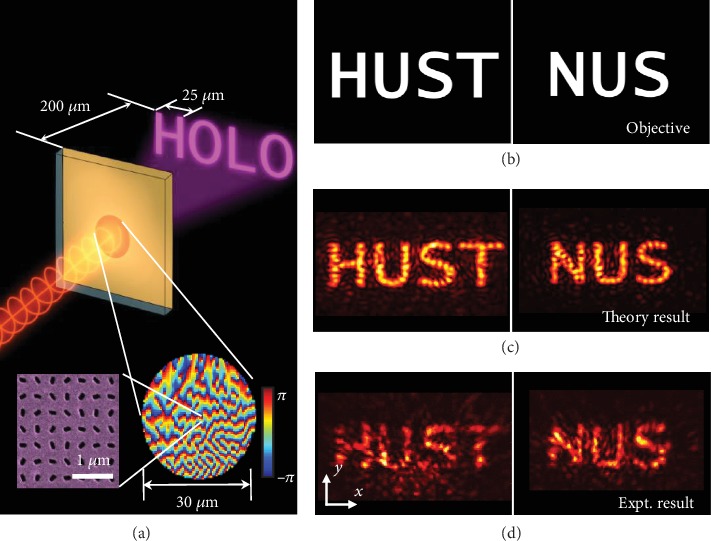
(a) Schematic illustration of holographic imaging of the SHG beam under a LCP fundamental beam in the Fresnel region; the inset shows the whole SH phase distribution for the SH hologram “HUST” and SEM image of the Au metasurface (partial). (b–d) The objective, theoretical, and experimental images of holographic imaging.

Such a nonlinear integrated interface also allows holographic imaging, as schematically illustrated in Figure 6(a), where the circle with a diameter of 30 *μ*m indicates the Au nanohole array. Under a LCP fundamental beam, the LCP component of the emitted SHG beam from the WS_2_ monolayer can be imaged in the Fresnel region. The image plane is designed to be 200 *μ*m away from the sample, and each letter in the pattern is 25 *μ*m wide. The designed objective images are the abbreviations for “Huazhong University of Science and Technology” and “National University of Singapore” (Figure 6(b)). The phase plane can be generated by an iterative method (further details are available in Figure S5 in SI). The theoretical images calculated by Fresnel-Kirchhoff's diffraction formula and the measured experimental images are shown in Figures 6(c) and 6(d), respectively. The intensity distributions of the measured images match the theory very well.

In conclusion, we have proposed and experimentally demonstrated a new platform for full control of the nonlinear wavefront of SHG based on the Au-TMDC nonlinear optical interface. The hybrid nonlinear interface hybridizes an Au nanosieve with a WS_2_ monolayer, and the former acts as an unprecedentedly strong and precise “spatial light modulator” down to the pixel size of a hundred nanometers for the latter. Our results prove that multifunctional SHG control, such as SH beam steering; versatile polarization control; dynamic OAM generation; and holography are enabled, together with large effective second-order nonlinear susceptibility of ~25 nm/V. It provides a strategy for the efficient generation and manipulation of SHG signals. From an application viewpoint, this control enables miniaturized, high-performance nonlinear optical devices that have thickness in the nanometer scale range. This therefore opens up a wide range of opportunities to realize ultrathin, ultracompact nonlinear optical devices, such as beam splitting, versatile polarization control, and free-space quantum communications.

## 2. Methods

### 2.1. Sample Fabrication

1. TheWS_2_ monolayers were fabricated on a sapphire substrate (SixCarbon Technology, Shenzhen). The solution of polymethyl methacrylate (PMMA, 2 wt%, Sigma-Aldrich) was drop-coated on the WS_2_-sapphire substrate. It was placed at room temperature for 1.5 h and then baked at 120°C for 0.5 h. The WS_2_-sapphire substrate was immersed in NaOH aqueous solution (3 mol/L) at room temperature for more than 4 h to etch the sapphire surface, and PMMA-WS_2_ films can be naturally lifted off from the sapphire substrate. The PMMA-WS_2_ films were fished out with a glass slide and immersed in deionized water for 3 times in order to remove the residual NaOH solution
2. A 60 nm thick gold film with a 5 nm thick Cr adhesion layer was deposited on a quartz substrate by using an e-beam evaporator. Then, the Au nanohole array with different arrangements was milled by the focused ion beam milling method (FIB, FEI Versa 3D)
3. With the help of a motorized stage-controlled needle, the PMMA-WS_2_ films were fished out by the fabricated metasurfaces, and then they were aligned quickly with the Au metasurface under the microscope before the water was dry. Finally, they were baked at 120°C for 1 h to improve the combination of the WS_2_ monolayers and the Au nanosieve and immersed in acetone for 3 times to remove the PMMA film

### 2.2. SHG Characterization

A mode-locked Ti-sapphire femtosecond laser centered at 810 nm (Vitara Coherent, 8 fs and 80 MHz) was used for the fundamental beam source. The polarization was adjusted by a quarter-wave plate (WPQ05M-808, Thorlabs). A home-built optical system was used to measure an emitted SH signal under an excitation of a plane-wave laser. The fundamental beam was focused by an 8 cm lens or an objective (Olympus, 10x and 0.25 NA) to generate different sizes of focal spots. The emitted signal was collected by an objective lens (Olympus, 40x and 0.65 NA), filtered spectrally, and imported to a CMOS camera (Prime 95B, Photometrics) or to a spectrometer (Acton 2500i with Pixis CCD camera, Princeton Instruments) through a fiber. The emitted SH signal was extracted by a Glan-laser polarizer with a quarter-wave plate at the SH wavelength (GL10-A and WPQ05M-405, Thorlabs). To measure the spatial intensity of the SH signals, we captured the SHG images at the different planes from 0 *μ*m to 200 *μ*m along the *z*-axis with a step-size of 0.5 *μ*m.

## Additional Points

*Additional Information*. Supporting information is available.

## Supplementary Materials

Supplementary Materials Figure S1: the schematic sketch of the experiment setup for SHG characterization. Figure S2: the emitted SHG beams along the *z*-axis from the same sample at *α* = 10° under the excitation of the LP and the RCP fundamental beam. Figure S3: numerically stimulated phase plates for the OAM generations shown in Figure 5 in the manuscript. Figure S4: determination of the topological charge of OAM. Figure S5: generative process of phase plates of holography.
