# Supplementary material for: Structuring Nonlinear Wavefront Emitted from Monolayer Transition-Metal Dichalcogenides
Source: Research (Wash D C). 2020 Apr 5;2020:9085782. doi: 10.34133/2020/9085782 (PMC7163797; doi:10.34133/2020/9085782)
Supplement: Supplementary Materials — Figure S1: the schematic sketch of the experiment setup for SHG characterization. Figure S2: the emitted SHG beams along the z-axis from the same sample at α = 10° under the excitation of the LP and the RCP fundamental beam. Figure S3: numerically stimulated phase plates for the OAM generations shown in Figure 5 in the manuscript. Figure S4: determination of the topological charge of OAM. Figure S5: generative process of phase plates of holography. [file 9085782.f1.docx]

**Structuring Nonlinear Wavefront Emitted from Monolayer Transition-Metal Dichalcogenides**

*Xuanmiao Hong^1†^, Guangwei Hu^2,3†^, Wenchao Zhao^1†^, Kai Wang^1*^, Shang Sun^2^, Rui Zhu^2,5^, Jing Wu^4^, Weiwei Liu^1^, Loh Kian Ping^6^, Andrew Thye Shen Wee^5,7^, Bing Wang^1^, Andrea Alù^3*^, Cheng-Wei Qiu^2^* and Peixiang Lu^1,8^**

^1^Wuhan National Laboratory for Optoelectronics and School of Physics, Huazhong University of Science and Technology, Wuhan 430074, China

^2^Department of Electrical and Computer Engineering, National University of Singapore, 4 Engineering Drive 3, Singapore 117583, Singapore.

^3^Advanced Science Research Center, City University of New York, New York 10031, USA.

^4^Institute of Materials Research and Engineering, A*STAR (Agency for Science, Technology and Research), 2 Fusionopolis Way, Innovis, #08-03, 138634, Singapore

^5^Department of Physics, National University of Singapore, 2 Science Drive 3, Singapore, 117551 Singapore

^6^Department of Chemistry National University of Singapore 3 Science Drive 3, Singapore 17543, Singapore

^7^Centre for Advanced 2D Materials, National University of Singapore, Block S14, 6 Science Drive 2, Singapore, 117546 Singapore

^8^Hubei Key Laboratory of Optical information and Pattern Recognition, Wuhan Institute of Technology, Wuhan 430205, China

^†^These authors contributed equally: Xuanmiao Hong, Guangwei Hu and Wenchao Zhao

*Corresponding authors:

Kai Wang, [kale_wong@hust.edu.cn](mailto:kale_wong@hust.edu.cn)

Andrea Alù, [aalu@gc.cuny.edu](mailto:aalu@gc.cuny.edu)

Cheng-Wei Qiu, [chengwei.qiu@nus.edu.sg](mailto:chengwei.qiu@nus.edu.sg)

Peixiang Lu, [lupeixiang@hust.edu.cn](mailto:lupeixiang@hust.edu.cn)

**
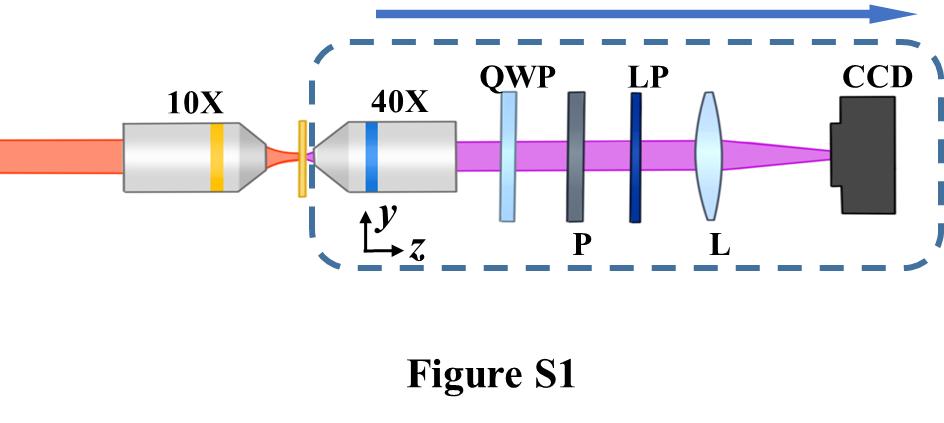
**

**Fig. S1** The schematic sketch of the experiment setup for SHG characterization. QWP: quarter-wave plate; P: Glan-laser polarizer; LP: 720-nm long pass filter; L: lens. The femtosecond laser centered at 810 nm was used for the fundamental beam source. The polarization was adjusted by a quarter-wave plate(not drawn). The fundamental beam was focused by an 8-cm lens or an objective to generate different sizes of focal spots. The emitted signal was collected by an objective lens, and imported to a CMOS camera or to a spectrometer through a fiber. The emitted SH signal was extracted by a Glan-laser polarizer with a quarter-wave plate at the SH wavelength. To measure the spatial intensity of the SH signals, we captured the SHG images at the different planes from 0 μm to 200 μm along z-axis with a step-size of 0.5 μm. For that , the whole collection system was put on a displacement platform moving along the z axis.

**
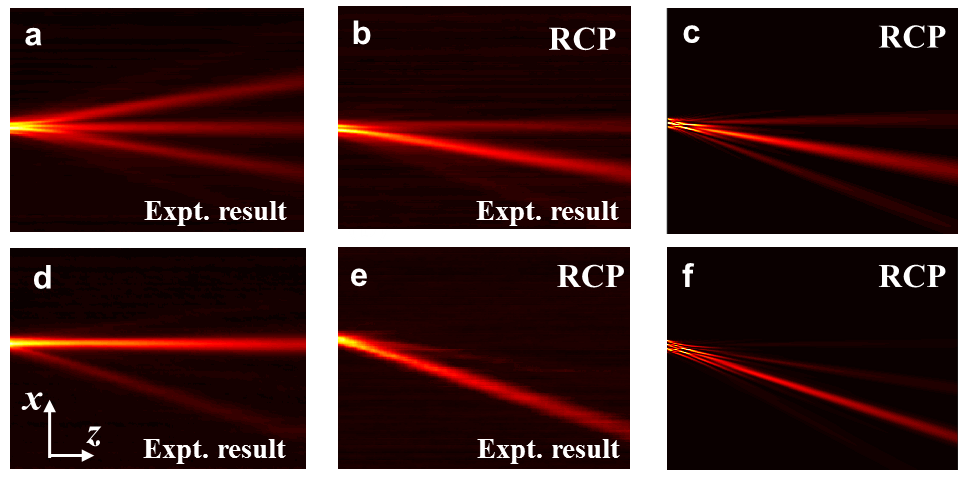
**

**Fig. S2** The emitted SHG beams along *z*-axis from the same sample at $\alpha=10^{\circ}$under the excitation of LP and RCP fundamental beam. **a** and **d.** the measured SHG beams under a excitation of LP and RCP fundamental beam, respectively. **b** and **e.** the extracted RCP components of the results from **a** and **d**, respectively. It can be observed that the 1^st^ order deflected beam under a RCP excitation is measured to be $20^{\circ}$, which is a double value of that under a LP excitation. **c** and **f** the numerical simulated results related to the experimental results in **b** and **e**, respectively.


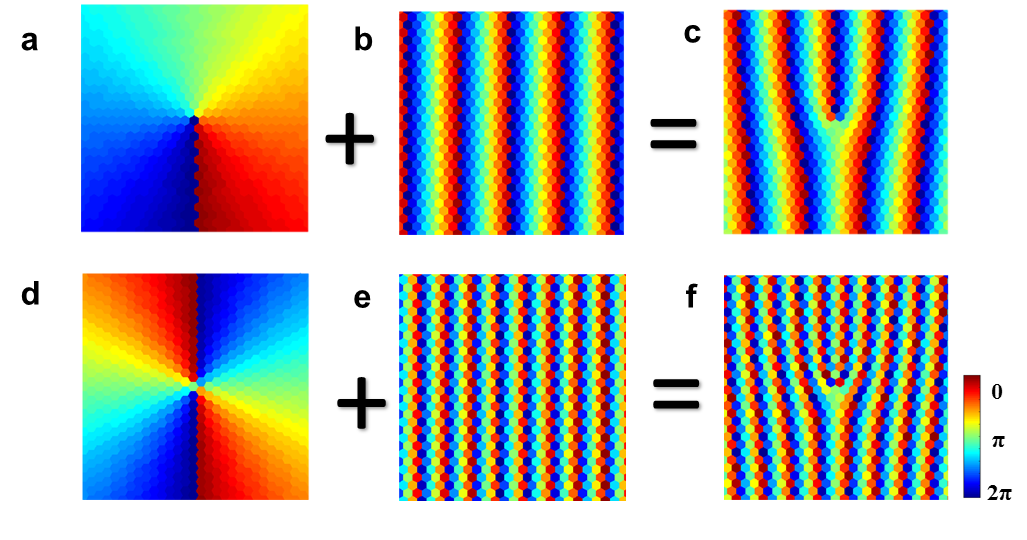
**Fig. S3** Numerically stimulated phase plates for OAM generations shown in Figure 5 in manuscript. **a** and **b.** a spiral phase plate ($n=1$) and the gradient phase plate under a LP excitation fundamental beam, respectively. **c.** the superposition of the phase plates in **a** and **b**, indicating a fork dislocation pattern. **d** and **e**. a spiral phase plate and the gradient phase plate for the same sample under a RCP excitation, respectively. Importantly, both the topological charge in **d** and the phase gradient in **e** have doubled, compared with that in **a** and **b**. **f.** the superposition of the phase plates shown in **d** and **e**, indicating a fork dislocation pattern with a double stripes than that in **c**. It indicates that both topological charge and the deflection angle for 1^st^ order deflected beam under a RCP excitation are doubled than that under a LP excitation.


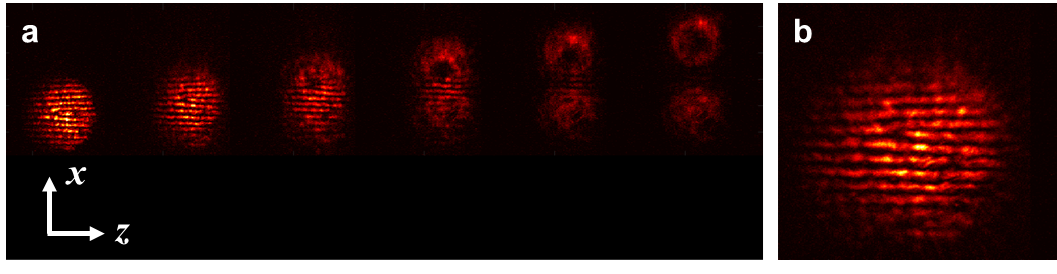


**Fig. S4** Determination of the topological charge of OAM. **a.** the extracted RCP components of the emitted SHG patterns from the sample under a LP excitation shown in Fig. 5a. The interval is 30 μm. **b.** the enlarged interference pattern captured at ***z***=30 μm. With a quarter-wave plate and a glan-laser polarizer, the only RCP components can be extracted from the experimental results shown in Fig. 5a in manuscript. As mentioned, the 0^th^ order beam is a LP beam without phase delay. And then, the residue is a RCP beam after filtering. Therefore, it can act as a good reference beam for determining the topological charge for deflected OAM beam. It can be clearly observed that +1^st^ order (OAM beam) and the 0^th^ order beam can interfere with each other, leading to an interference pattern shown in **b**. It shows a clear fork dislocation patterns which is moved with the OAM beam. The topological charge of the OAM beam can be determined to be 1 by the extra stripes of the interference pattern, which is consistent with the design. Moreover, the topological charge for +2^nd^ order deflected beam should be 2 in theory, but it is too weak to be measured in our experiment.

**
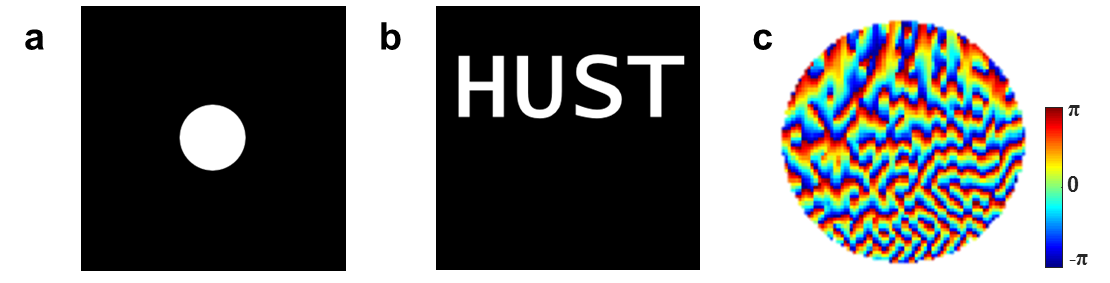
**

**Fig. S5** Generative process of phase plates of holography. **a** and **b**. The amplitude distribution of SHG on sample and image plane. Here, the radius of the circle shown in **a** is 30 μm and the pattern width in **b** is about 100 μm. Note that the pattern was not placed in the center in order to avoid interference from signal along the *z*-axis. **c.** The calculated phase plates. First, a light field was created with random phase distribution and an amplitude distribution shown in **a**. Then calculate the light field on the image plane (propagated ~200 μm) and replace its amplitude distribution with **b**. Afterwards, calculate the light field on the sample plane (back-propagation 200 μm from image plane) and replace its amplitude distribution with **a**. The process was repeated 30 times. The phase distribution on the sample plane was the phase plates of the pattern in **b**.

1. **Phase control with the geometric phase.**

The nanohole can be considered as a polaroid. The jones’ matrix can be calculated by $J(\theta)=R(-\theta)\left[ \begin{matrix} 1 & 0 \\ 0 & 0 \end{matrix} \right]R(\theta)$, where $\theta$ is the rotation angle of nanohole, and $R\left( \theta\right)=\left( \begin{matrix} \cos\theta& \sin\theta\\ -sin\theta& \cos\theta\end{matrix} \right)$ is the rotation matrix. If the incident filed is y-polarized $\left[ \begin{matrix} 1 \\ 0 \end{matrix} \right]$, then the transmitted field will be:

$\left[ \begin{matrix} E_{x}\left( \omega\right) \\ E_{y}(\omega) \end{matrix} \right]=\left[ \begin{matrix} \cos^{2}\left( \theta\right) \\ \sin\left( \theta\right)\cos\left( \theta\right) \end{matrix} \right]$ (1)

Then it can be decomposed into:

$\left[ \begin{matrix} E_{x}(\omega) \\ E_{y}(\omega) \end{matrix} \right]=\frac{1}{4}\left\{ 2\left[ \begin{matrix} 1 \\ 0 \end{matrix} \right]+e^{i2\theta}\left[ \begin{matrix} 1 \\ -i \end{matrix} \right]+e^{-i2\theta}\left[ \begin{matrix} 1 \\ i \end{matrix} \right] \right\}$ (3)

It indicates that there are three components: a LP field without phase delay and a RCP and LCP field with a phase factor ±$e^{i2\theta}$. For monolayer WS2 with the $D_{3h} \left( \bar{6}m2 \right)$ point group symmetry, and assume that the symmetry axis of monolayer WS2 is parallel to the x-axis. The second-harmonic susceptibility is:

$\chi_{xxx}^{\left( 2 \right)}=-\chi_{xyy}^{\left( 2 \right)}=-\chi_{yxy}^{\left( 2 \right)}=-\chi_{yyx}^{\left( 2 \right)}=\chi^{\left( 2 \right)}$ (4)

The nonlinear signal can be calculated as

$P_{x}\left( 2\omega\right)\propto\chi_{xxx}^{\left( 2 \right)}\left[ E_{x}\left( \omega\right) \right]^{2}+\chi_{xyy}^{\left( 2 \right)}\left[ E_{y}\left( \omega\right) \right]^{2}$ (5)

$P_{y}\left( 2\omega\right)\propto2\chi_{xyx}^{\left( 2 \right)}E_{x}\left( \omega\right)E_{y}\left( \omega\right)$ (6)

We can have:

$E_{x}\left( 2\omega\right)\propto\chi^{\left( 2 \right)}\left\{ \left[ E_{x}\left( \omega\right) \right]^{2}-\left[ E_{y}\left( \omega\right) \right]^{2} \right\}$ (7)

$E_{y}\left( 2\omega\right)\propto2\chi_{xyx}^{\left( 2 \right)}E_{x}\left( \omega\right)E_{y}\left( \omega\right)$ (8)

And then it can be decomposed into:

$\left[ \begin{matrix} E_{x}\left( 2\omega\right) \\ E_{y}\left( 2\omega\right) \end{matrix} \right]=\frac{1}{8}\left\{ (e^{-i4\theta}+2e^{-i2\theta})\left[ \begin{matrix} 1 \\ -i \end{matrix} \right]+2\left[ \begin{matrix} 1 \\ 0 \end{matrix} \right]+(e^{i4\theta}+2e^{i2\theta})\left[ \begin{matrix} 1 \\ i \end{matrix} \right] \right\}$ (9)

It indicates that the emitted SHG have five components with different phase delay and polarization. Therefore, these components can be separated spatially with different defection angles. Specifically, the LP beam without phase delay is propagated along ***z***-axis, the other components are deflected. The local-field of SHG from each nanohole can be calculated, and the summation is the total field of a sample. Fig. 4c,f show the experimental and simulated RCP component of the emitted SHG beams at $\alpha=10^{\circ}$. It can be observed a strong 1^st^ order deflected beam at 10° in both figures. Moreover, two weak beams at 0° and 20° can also be considered as the diffraction signals of the strong signal because of the amplitude modulation of the sample. We also prepare samples with deflection angles of 5° and 15°. It can be observed that the defection angles of the 1^st^ order deflected beams of each sample agree with the designs well. It is worth noting that the 2^nd^ order deflected beam with a twice of designed value of deflection angle can only be clearly observed in experimental in Figure 4b, which may ascribed to the much weaker beam intensity and a limited signal collected efficiency.

Alternatively, these samples can also work under a CP excitation. With a RCP incident field $\frac{1}{\sqrt{2}}\left[ \begin{matrix} 1 \\ i \end{matrix} \right]$, the transmitted field can be written as:

$\left[ \begin{matrix} E_{x}(\omega) \\ E_{y}(\omega) \end{matrix} \right]=\frac{1}{2}\left\{ e^{i2\theta}\left[ \begin{matrix} 1 \\ -i \end{matrix} \right]+\left[ \begin{matrix} 1 \\ i \end{matrix} \right] \right\}$ (10)

With the second-harmonic susceptibility the nonlinear signal can be calculated as

$\left[ \begin{matrix} E_{x}\left( 2\omega\right) \\ E_{y}\left( 2\omega\right) \end{matrix} \right]=\frac{1}{2}\left\{ \left[ \begin{matrix} 1 \\ -i \end{matrix} \right]+e^{i4\theta}\left[ \begin{matrix} 1 \\ i \end{matrix} \right] \right\}$ (11)

Figure S2 shows the emitted SHG beams from the same sample at α=10° under the excitation of LP and RCP fundamental beam. Under the excitation of RCP, the 0^th^ order beam was LCP beam without phase delay. The +1^st^ order was RCP beam with phase factor of $e^{i4\theta}$. Note that it has the same deflection angle with that of the +2^nd^ order beam under a LP excitation because it has the same phase factor of $e^{i4\theta}$.

1. **The comparison with conventional nonlinear metasurface.**

In this part, we would highlight the difference between the conventional nonlinear metasurface and our synthetic metasurface, which hopefully could address the novel physics in this work. According to those seminal works**^1-5^**, two important conclusions based on spin-orbit coupling in nonlinear light-matter interaction have been reached and adopted to design the various nonlinear meta-devices. The first one is regarding to the nonlinear selection rules of the metasurface with meta-atoms of *m*-fold rotational symmetry, which is only harmonic orders of $n=lm\pm1$ are allowed upon the CP pumping, where $l$ is an integer and that the ‘$+$’ and ‘$-$’ signs correspond to harmonic generation of the same and opposite circular polarization, respectively. The second one is regarding to the nonlinear geometric phase (PB-phase). The nonlinear field will carry the geometric phases $(n-1)\sigma\theta$ or $(n+1)\sigma\theta$ with the same or opposite circular polarization to that of the fundamental wave, respectively. We would like to point out the fact neither of them apply to our synthetic metasurface due to the difference of our mechanism.

In our synthetic metasurface, $n=2$ and we use “+” because it is the same spin of SH that carries the geometric phase. If the symmetry of rectangular nanohole is considered, then m=2, which should not allow the SHG since $2=2l+1$ cannot be satisfied with the integer *l*. If the symmetry of monolayer WS_2_ is considered, then m=3, which could not happen either since $2=3l+1$ cannot be satisfied with the integer *l*. Moreover, in our system the geometric phase under the CP pumping is the 4$\theta$, which doesn’t correspond to any nonlinear geometric phase given above. The fundamental reason behind is the physics of SHG. While SHG in traditional nonlinear metasurface are induced by the structure of the nanostructure with additional spin-orbit coupling in nonlinear light-matter interaction, the SHG in our system are generated by the natural materials without spin-orbit coupling. Thus, from the perspective in the discussion above, we believe our work could provide new approaches and broad inspiration for the community of nonlinear metasurface.

1. **The comparison of nonlinear performance.**

| Nonlinear medium | η_SHG_ | χ(2)_eff_ | SHG wavelength |
| --- | --- | --- | --- |
| **metasurfaces** | | | |
| Nonlinear photonic crystals**^4^** | 5.4*10^-10^ | 18 pm/V | 600 nm |
| Multiple quantum well**^5^** | 2*10^-6^ | 54 nm/V | 8 μm |
| MQW nanoparticles**^6^** | 8.8*10^-4^ | 420 nm∕V | 9.85 μm |
| Nanoantenna**^7^** | 4.4*10^-6^ | 25 pm/V | 545 nm |
| Nanoparticles with gap**^8^** | 1.8*10^-7^ | 6.1 pm/V | 439 nm |
| Nanocup**^9^** | 1.8*10^-9^ | 3.2 pm/V | 400 nm |
| C3 plasmonics meta-atoms**^10^** | 4.51*10^–9^ |  | 550 nm |
| Nanoantenna**^11^** | 4*10^−7^ | 40 pm/V | 750 nm |
| Nanoantenna**^12^** | 5*10^-10^ |  | 780 nm |
| **2D materials** | | | |
| MoS_2_ monolayer**^13^** |  | 5 nm/V | 410 nm |
| WS_2_ monolayer**^14^** |  | 4.5 nm/V | 416 nm |

**References**

1. Li G, Chen S, Pholchai N, Reineke B, Wong PW, Pun EY, et al. Continuous control of the nonlinearity phase for harmonic generations. Nat Mater 2015, 14(6): 607-612.
2. Li G, Zhang S, Zentgraf T. Nonlinear photonic metasurfaces. Nature Reviews Materials 2017, 2(5): 17010.
3. Krasnok A, Tymchenko M, Alù A. Nonlinear metasurfaces: a paradigm shift in nonlinear optics. Materials Today 2018, 21(1): 8-21
4. Segal, N.; Kerenzur, S.; Hendler, N.; Ellenbogen, T. Nat Photonics 2015, 9, (3).
5. Tymchenko, M.; Gomezdiaz, J. S.; Lee, J.; Nookala, N.; Belkin, M. A.; Alù, A. Phys Rev Lett 2015, 115, (20), 207403.
6. Alu, A.; Demmerle, F.; Shvets, G.; Boehm, G.; Gomezdiaz, J. S.; Lee, J.; Lai, K.; Amann, M. C.; Belkin, M.; Tymchenko, M. Optica 2016, 3, (3), 283.
7. Chen, P. Y.; Argyropoulos, C.; D'Aguanno, G.; Alu, A. Acs Photonics 2015, 2, (8), 1000-1006.
8. Dong, Z.; Asbahi, M.; Lin, J.; Zhu, D.; Wang, Y. M.; Hippalgaonkar, K.; Chu, H.-S.; Goh, W. P.; Wang, F.; Huang, Z.; Yang, J. K. W. Nano Lett 2015, 15, (9), 5976-5981.
9. Zhang, Y.; Grady, N. K.; Ayalaorozco, C.; Halas, N. J. Nano Lett 2011, 11, (12), 5519-5523.
10. Li, G.; Wu, L.; Li, K. F.; Chen, S.; Schlickriede, C.; Xu, Z.; Huang, S.; Li, W. D.; Liu, Y. J.; Pun, E. Y. Nano Lett 2017, 17, (12), 7974–7979.
11. Gennaro, S. D.; Rahmani, M.; Giannini, V.; Aouani, H.; Sidiropoulos, T. P. H.; Navarro-Cia, M.; Maier, S. A.; Oulton, R. F. Nano Lett 2016, 16, (8), 5278-5285.
12. Celebrano, M.; Wu, X. F.; Baselli, M.; Grossmann, S.; Biagioni, P.; Locatelli, A.; De Angelis, C.; Cerullo, G.; Osellame, R.; Hecht, B.; Duo, L.; Ciccacci, F.; Finazzi, M. Nat Nanotechnol 2015, 10, (5), 412-417.
13. Kumar, N.; Najmaei, S.; Cui, Q.; Ceballos, F.; Ajayan, P. M.; Lou, J.; Zhao, H. Physical Review B Condensed Matter 2013, 87, (16), 97-101.
14. Janisch, C.; Wang, Y. X.; Ma, D.; Mehta, N.; Elias, A. L.; Perea-Lopez, N.; Terrones, M.; Crespi, V.; Liu, Z. W. Sci Rep-Uk 2014, 4.
